# Dietary Supplementation of Leucine in Premating Diet Improves the Within-Litter Birth Weight Uniformity, Antioxidative Capability, and Immune Function of Primiparous SD Rats

**DOI:** 10.1155/2018/1523147

**Published:** 2018-04-18

**Authors:** Ting Liu, Bin Zuo, Wei Wang, Shilan Wang, Junjun Wang

**Affiliations:** State Key Laboratory of Animal Nutrition, College of Animal Science and Technology, China Agricultural University, Beijing 100193, China

## Abstract

The high within-litter birth weight variation has become a big issue in multiparous animals. The present study was conducted to investigate the effects of leucine supplementation in premating diet on the reproductive performance, maternal antioxidative capability, and immune function in primiparous rats. Six-week-old female SD rats were assigned to basal diet or 0.6% leucine supplemented diet for two weeks. After mating during the eighth week of age, the rats were fed with regular gestation diet. Maternal blood samples were collected on the day before mating (day −1) and day 7 and day 20 of pregnancy, while ovaries and uteruses were obtained on day −1 and on day 7, respectively. The results indicate that, compared with control group, within-litter birth weight variation was significantly decreased, while birth weights were significantly increased in the leucine group (*P* < 0.01). Also, leucine improved the embryo distribution uniformity and the number of implantation sites in uterine. The ovarian gene expressions of* LHR*,* CYP19A1*, and* VEGFA* were upregulated, while* Mucin-1* was decreased significantly (*P* < 0.05). Leucine also increased the maternal antioxidant capacity and immune function. Conclusively, leucine supplementation in premating diet could improve the reproductive performance, which could be attributed to the improved oxidative and immune status.

## 1. Introduction

Over the past few decades, advances in selecting and breeding have significantly increased the litter sizes in modern high-producing sows [1]. However, this increase in litter size has coincided with an enlarged number of growth retarded piglets and increased preweaning mortality and heterogeneity at ten weeks of age [2, 3], and these losses can be partly attributed to the increased proportion of piglets of low birth weight. Importantly, the increased preweaning mortality is likely the result of the high within-litter birth weight variation [1, 4]. During the peri-implantation period, conceptuses can show different stages in elongation process in uterus [5], indicating that the diversity of developmental ability of conceptuses may lead to the variation in fetal growth [6] and decrease the birth weight uniformity of littermates. Considering the fact that the oocyte quality is the main factor affecting early embryonic survival and fetal development [7], nutritional interventions during the follicular growth period may decrease within-litter birth weight variation. In addition, maternal physiology condition, especially antioxidative capability and immune function, acts as a potential influence factor for the pregnancy results. It has been reported that a balance between reactive oxygen species (ROS) and antioxidant not only plays a great role in hormone secretion of ovary, but also benefits oocyte maturation and quality [8–10]. Also, some other researches indicated that maternal immune system influences reproductive event, particularly around the time of conception and embryo implantation [11], and immune dysregulation is implicated in adverse perinatal health outcomes [12].

Leucine, like other essential nutrients required for maintaining life in rodent [13], needs to be supplemented in diet because animals cannot synthesize it by themselves [14]. Leucine metabolism has been largely reported to be closely associated with early-state embryo development by activating the mTOR signal pathway [15, 16]. Besides, as a prerequisite for fetus growth, leucine has an important function in blastocyst development, which may further contribute to embryo implantation [17]. It has been proven that leucine could trigger trophectoderm motility and upregulate blastocyst outgrowth in the peri-implantation period* in vitro* [18, 19], eventually improving the success rate of mouse embryonic implantation. However, to our knowledge, the effects of leucine supplementation during the follicular growth period on the outcome of pregnancy are rarely investigated. We hypothesized that leucine supplementation during preconception period facilitates oocyte maturation and promotes embryonic implantation uniformity during the peri-implantation period, ultimately increasing the littermate birth weight uniformity. To this aim the present study was conducted to investigate the effects of leucine administration in premating diet on within-litter birth weight variation, maternal antioxidative capability, and immune function in primiparous SD rats.

## 2. Materials and Methods

### 2.1. Animals

Sprague-Dawley female rats aged five weeks were purchased from Sibeifu Inc (Beijing, China). Upon arrival, there was one week for adaptation. They were housed in individual cages in a climatized environment at 23°C in a room with a 12 h light/12 h dark cycle (lights on from 8:00 am to 8:00 pm) and were given free access to water and feed. All animal procedures were approved by the China Agricultural University Institution Animal Care and Use Committee.

### 2.2. Diet and Experimental Design

The experimental groups contain a control group (basal diet for adult rodent) and a leucine group (basal diet supplemented with 0.6% l-Leucine), and the treatment lasted for two weeks from six to eight weeks of age. After two weeks of leucine administration, each female rat was cocaged with one male SD rat proven fertile overnight, and the presence of spermatozoa in the vaginal smear in the next morning was defined as day 1 of pregnancy. Females that did not mate were reintroduced to males and daily leucine manipulation was repeated until mating was achieved. Mating had been attempted for a maximum of 2 nights, with those that did not mate being excluded from the study.

### 2.3. Plasma and Tissue Sampling

At the end of the leucine administration, the day before mating was defined as day −1, 10 dams from each group were anesthetized with sodium pentobarbital and then sacrificed 5 min later. Ovary and uterus were immediately snap-frozen in liquid nitrogen and stored in −80°C for quantification of gene expression and determination of the immune function. Liver tissue was collected for antioxidant capacity analysis. On day 7 of pregnancy, at which point the embryo establishes contact with the uterine endothelium, 10 dams from each group were anesthetized and killed. After exposing the uterine horns, the implantation sites were counted and collected. In addition, considering the effect of birth order, specifically the uncertainty of delivery time and the intake of colostrum on fetal body weight, the remaining pregnant rats (*n* = 12/group) were sacrificed to evaluate the within-litter birth weight variation on day 20 of gestation (duration of pregnancy is 21–23 days). On day −1 and days 7 and 20 of pregnancy, blood was collected from the vena cava into heparinized vacuum blood tube for analysis of amino acids, hormone, and metabolites concentration. Blood samples were centrifuged at 3,000 rpm for 15 min and stored at −20°C.

### 2.4. Biochemical Analysis

Plasma concentrations of insulin, insulin-like growth factor 1 (IGF-1), estradiol, progesterone, luteinizing hormone (LH), and follicle stimulating hormone (FSH) were analyzed by radio immunoassay kits. The levels of plasma immunoglobulins (IgA, IgG, and IgM), interleukins (IL-1*β* and IL-6), and tumor necrosis factor *α* (TNF *α*) were measured using commercial ELISA kits for rats. Redox-status-related parameters including malondialdehyde (MDA), superoxide dismutase (SOD), glutathione peroxidase (GSH-Px), and catalase (CAT) in liver were determined using enzyme immunoassay kits. All the assay kits were purchased from Sino-UK Inc (Beijing, China) and processed following the instructions provided by the manufacturer. Concentrations of amino acids in plasma were determined by a high-performance liquid chromatography (HPLC) involving precolumn derivatization with* o*-phthaldialdehyde [20, 21].

### 2.5. RNA Extraction and Quantitative Real-Time PCR Analysis

The RNA extraction from implantation sites was performed using the TRIzol reagent (CWBIO, China) according to the manufacturer's protocol, and the total RNA concentration and purity were evaluated using NanoDrop spectrophotometer (Thermo Scientific, USA). A total of 1.0 *μ*g RNA was reverse transcribed to complementary DNA (cDNA) using RevertAid 1st Strand cDNA Synthesis Kit (Thermo, USA). Then cDNA was used for amplifying by specific primers (Supplementary Table 1) using SYBR Green (Takara, Japan) with an Applied Biosystems 7500 Real-Time PCR System (ABI, USA). The PCR system consisted of 5.0 *μ*L of SYBR Green qPCR mix, 1.0 *μ*L of cDNA, 1.0 *μ*L of primer pairs (0.5 *μ*L forward and 0.5 *μ*L reverse), and 3.0 *μ*L of double distilled water with a total volume of 10.0 *μ*L. The protocols for gene quantification included a denaturation program (10 min at 95°C) and amplification and quantification program repeated for 40 cycles (15 s at 95°C, 60 s at 60°C), followed by a melting curve program at 60–95°C with a heating rate of 0.1°C per second and continuous fluorescence measurement. Data were analyzed using the 2^−ΔΔct^ method as described [22].

### 2.6. Statistical Analysis

Values were expressed as means ± SEM. Effects of leucine on the outcome of pregnancy were analyzed using a Student's unpaired *t*-test (GraphPad Prism version 7.0). *P* < 0.05 was considered significantly different, and 0.05 < *P* < 0.1 was considered a tendency. 

## 3. Results

### 3.1. Reproductive Performance

**Figure 1:**
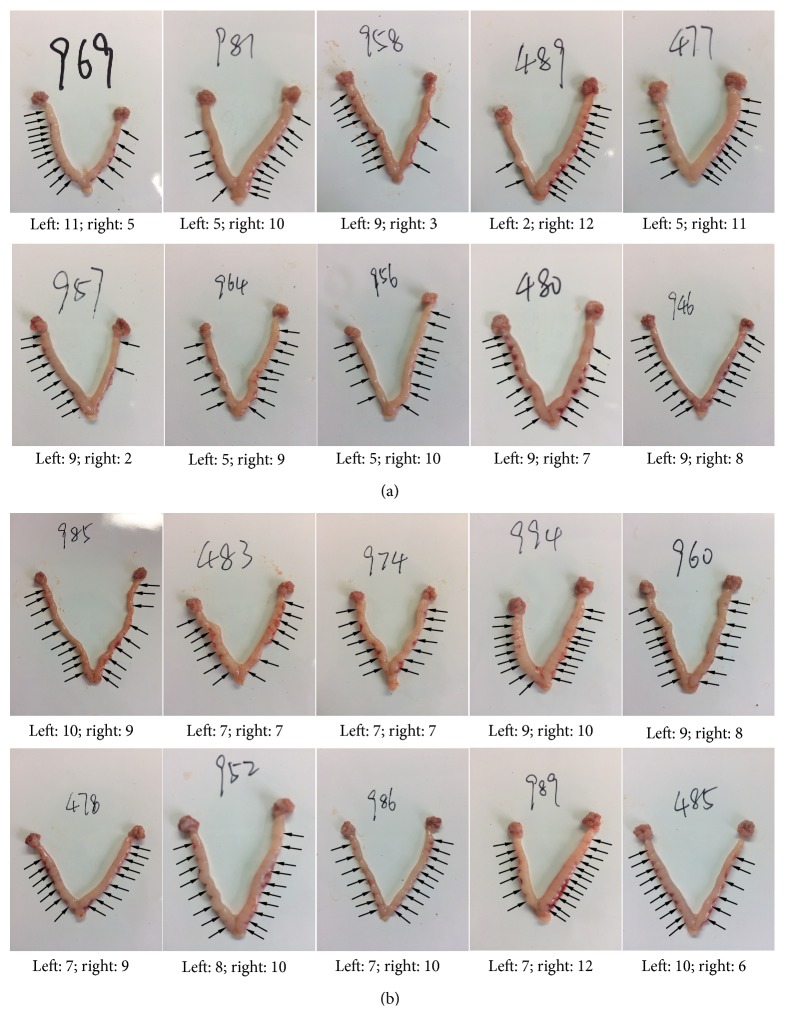
Effect of dietary leucine supplementation on implantation on day 7 of pregnancy in SD rats. Implantation site distributions in control (a) and leucine (b) group.

In the present study, there is no statistical difference in average daily feed intake between the control and leucine supplementation group (Table 1). Fetal birth weight was indicated as body weight at late gestation (day 20 of pregnancy). Variation of within-litter birth weight was determined as a coefficient of littermate birth weight. The reproductive outcomes were presented in Table 2. In the leucine group, within-litter birth weight variation was significantly downregulated, compared to the control group (*P* < 0.05). At the same time, litter weight in the leucine supplemented group was higher (*P* < 0.01) than that in the control group. In addition, placental weight was significantly higher (*P* < 0.05) in the leucine group. There was no difference in litter size between the two groups. In the early gestational stage, embryonic implantation was investigated on day 7 of pregnancy, during which blastocysts establish contact with endometrium. There was no difference in total number of implantation sites (Table 3). Extreme embryo distribution in two uterine horns indicated by the number of implantation sites was only observed in the control group (Figure 1). No statistical difference was found in body weight on day −1 of pregnancy (Table 4), whereas the relative weight of uterus was higher (*P* < 0.01) in the leucine group when compared to the control group. Although the absolute weight of the ovary was higher (*P* < 0.05) in the leucine group, the relative weight did not differ between these two groups (Table 4).

### 3.2. Hormone Concentration

Maternal plasma reproductive hormone concentrations were presented in Table 5. Compared with the control group, both the IGF-1 and estradiol level were higher at all the tested time points during pregnancy (all *P* < 0.05) in the leucine group. In addition, insulin level was reduced (*P* < 0.05), while LH concentration was higher (*P* < 0.05) on day −1 of pregnancy, progesterone concentration was upregulated (*P* < 0.05) on day 7 of pregnancy, and FSH level was higher (*P* < 0.05) on day 20 of pregnancy by supplementing leucine in the diet.

### 3.3. Gene Expression

**Figure 2:**
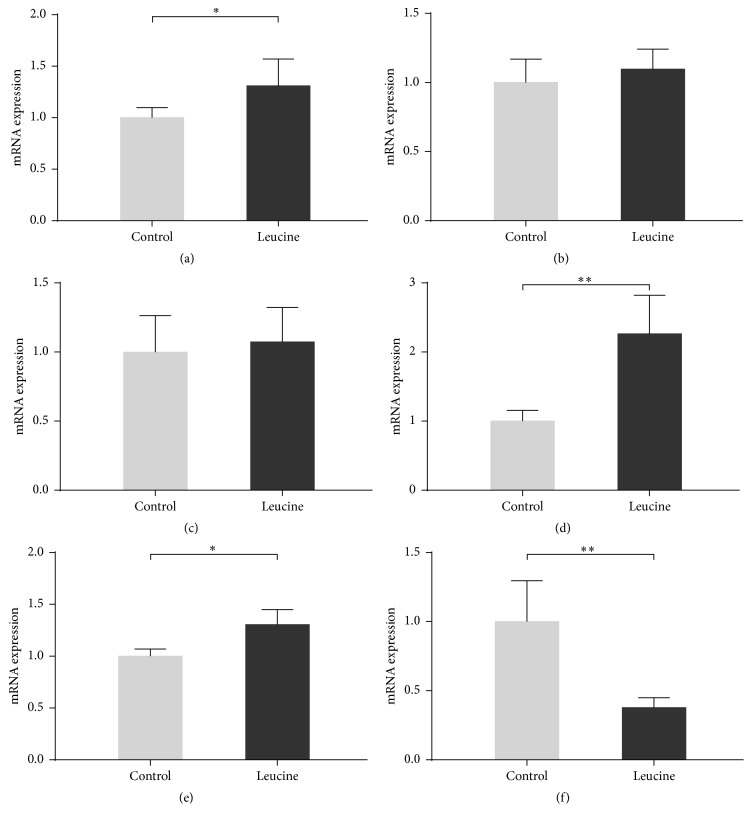
Effect of dietary leucine supplementation on the expression of gene involved in ovarian receptivity on day −1 of pregnancy in SD rats. The gene expression levels of* LHR* (a),* FSHR* (b),* CYP17A1* (c),* CYP19A1* (d),* VEGFA* (e), and* Mucin-1* (f) in ovary on day −1 of pregnancy were measured by quantitative real-time PCR. Data is expressed as mean ± standard error of means of nine replicates. ^*∗*^Labeled with significant difference (*P* < 0.05); ^*∗∗*^labeled with extreme difference (*P* < 0.01).

On day −1 of pregnancy, ovarian receptivity-related gene expressions were determined by quantitative real-time PCR. Leucine supplementation upregulated the expression of* LSHR* (Figure 2(a)),* CYP19A1* (Figure 2(d)), and* VEGFA* (Figure 2(e)), while it downregulated* Mucin-1* (Figure 2(f)). No statistical difference was found in expression of* FSHR* (Figure 2(b)) and* CYP17A1* (Figure 2(c)) between the two groups.

### 3.4. Antioxidative Capability

Effect of leucine on the antioxidant enzyme activity and oxidant products on day −1 of pregnancy were presented in Table 6. Leucine administration upregulated the level of SOD (63.02 ± 1.10 and 51.29 ± 2.60, leucine and control group, resp., *P* < 0.01), and it also upregulated CAT and GSH-Px concentrations in plasma (*P* < 0.05). Besides, the level of CAT in liver from the leucine group (58.66 ± 1.29) was higher than that in the control group (53.52 ± 3.20) (*P* < 0.05). There was no difference on MDA concentration in both plasma and liver.

### 3.5. Immune Function

**Figure 3:**
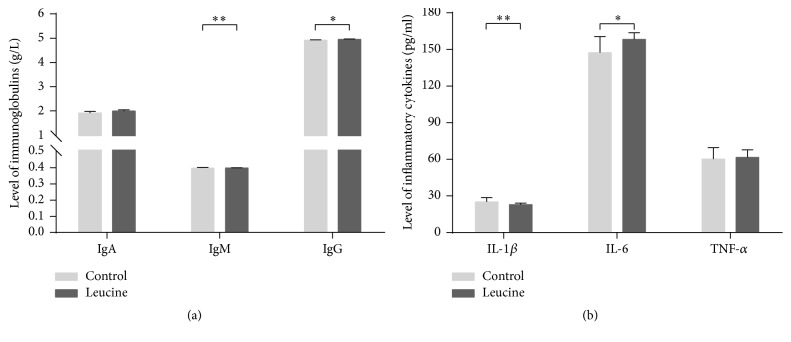
Effect of dietary leucine supplementation on the level of immunoglobulin (a) and inflammatory cytokines (b) in the plasma on day −1 of pregnancy in SD rats. Data is expressed as mean ± standard error of means of nine replicates. ^*∗*^Labeled with significant difference (*P* < 0.05); ^*∗∗*^labeled with extreme difference (*P* < 0.01).

There was a marked effect of leucine on the level of immunoglobulin (Figure 3(a)) and inflammatory cytokines in the plasma (Figure 3(b)). Supplementation of leucine upregulated the plasma concentrations of IgM, IL-1*β*, IgG, and IL-6 concentrations (*P* < 0.05) on day −1 of pregnancy. Dietary leucine administration did not affect the plasma level of IgA and TNF *α* on day −1 of pregnancy.

### 3.6. Amino Acids Profile

On day −1 of pregnancy, leucine supplementation significantly upregulated the plasma concentration of alanine, glutamine, arginine, leucine (*P* < 0.05), and aspartic acid (*P* < 0.01). Plasma phenylalanine and isoleucine showed a trend to increase (*P* < 0.10). But leucine had no effect on those of other measured amino acids, including cysteine, glycine, glutamic acids, methionine, lysine, tyrosine, proline, tryptophan, serine, threonine, asparagine, valine, and histidine, as compared with the control group (Table 7). On day 7 of pregnancy, only plasma aspartic acids showed a lower level (*P* < 0.05) than control group, while methionine concentrations had a trend to increase (*P* < 0.10) and the level of tryptophan trended to decrease (*P* < 0.10) in the leucine group (Table 7).

## 4. Discussion

Currently, special attention has been given to high variation of within-litter birth weight in modern prolific sows, which is characterized with a higher proportion of low birth weight newborns [23, 24] and their poor postnatal survival and subsequent growth performance [1]. Leucine has been largely reported to play key roles in oocyte maturation and early embryo development [19]. In the present study, our results demonstrate that leucine supplementation in the premating diet decreases the within-litter birth weight variation, and the increased embryonic implantation uniformity in two uterine horns and more appropriate strategies of amino acids utilization could partially explain the effect of leucine supplementation on neonatal outcomes. Besides, better body physiology condition, as shown by the increased antioxidative capability and immune function, may also contribute to improving the maternal phenotype.

One potential way to reduce within-litter variation of birth weight is nutritional interventions in premating period, because it has been demonstrated that diet composition fed before insemination can affect the distribution of fetal size within a litter [25]. In the current study, fetuses of leucine supplementation group show better within-litter birth weight uniformity than that in the control group, concomitant with increased litter weight. Consistent with the previous findings, it has been shown in pig that nutritional manipulation before and during the follicular phase seems to have the potential to decrease the within-litter variation in birth weight [26]. It can be speculated that dietary leucine supplementation had a positive function in premating period, likely during follicular growth phase. Specifically, the effects of leucine administration on within-litter birth weight variation could be attributed to its effect on follicle quality and/or oocyte quality [27]. However, as there was no difference in litter size, we suppose that the limited uterine space is another restricted factor of fetal development [28]. Although the absolute weight of the ovary was higher in the leucine group, the relative weight did not differ between these two groups. The higher absolute weight of ovary could indicate a better oocyte quality or larger follicular size in the leucine group. In addition, on maternal metabolism level, different plasma metabolites were found between the leucine and control groups. Dams in leucine group showed increased concentration of plasma estradiol accompanied by augment in FSH level, which may be indicator of an upregulation of ovarian response to gonadotropins.

Establishing contact with the endometrium is a challenge for blastocysts during the window of implantation, which requires both competent blastocyst and receptive uterus. However, manifest differences in blastocyst developmental competence lower the successful rate of implantation [5]. Abundant estradiol secreted from advanced embryos stimulates uterine secretions to their own benefit, however, which is detrimental to the less-developed littermate embryos [29, 30]; therefore, considerable embryonic loss occurs in the peri-implantation phase. Based on decreased within-litter birth weight variation in the leucine group, the effects of leucine on embryonic implantation were investigated on day 7 of pregnancy. And results have shown that as an aspect of severe asymmetrical distribution of embryos observed in the control group, it could result from absorption of less-competent blastocysts. Like late gestation phase in this study, in the early pregnancy, leucine increased the circulating progesterone concentration as well as IGF-1 and IGF-1-induced steroidogenesis by activating MAPK ERK1/2 phosphorylation [31], which could positively regulate progesterone and estradiol production in cultured rat granulosa cells [32].

Early results suggested that leucine was particularly important for initiation of embryo outgrowth [33]. González et al. [19] removed leucine from the culture medium resulting in quiescent embryos that fail to form trophoblastic outgrowths. In this study, we found higher concentration of estradiol and IGF-1 in rats treated with leucine at all the three-time points during pregnancy. The reason might be that estradiol is the major signal for maternal recognition of pregnancy, especially, which coincides with the time of concepts elongation [34, 35]. Meanwhile, the higher concentration of IGF-1 in the leucine group is in accordance with previous study that demonstrated that the development of follicles and oocytes is improved owing to higher concentration of IGF-1 [36]. Other researches demonstrated that higher IGF-1 level during the ovarian follicular phase reduced within-litter variation in birth weight in the subsequent litter [27, 37]. In addition, the level of LH was increased on day −1 of pregnancy in the leucine group. This could be explained by the fact that leucine administration has a positive feedback on the hypothalamus-pituitary axis leading to an increase in gonadotropins release which may act to modulate follicle development. On the other hand, during follicular growth, with the increasing level of LH, recruited follicles can increase the release of estradiol production [38] and therefore further follicular development.

We further evaluated the gene expression which is related to the follicular growth and oocyte maturation. Correspondingly, leucine upregulated expressions of* LHR*,* CYP19A1*, and* VEGFA* genes and downregulated levels of* Mucin-1*.* CYP19A1* is a key gene for steroid synthesizing and plays a critical role in follicle recruitment. In another study,* CYP19A1* mRNA had a better expression in the larger follicles (6–9 mm in diameter), compared with some follicles of 4–6 mm [39]. Increasing results suggest that* VEGFA* is regarded as a useful marker of follicle quality [40]. Direct injection of* VEGF* gene fragments into the ovaries of rats not only increased* VEGF* mRNA and protein in the ovary, but also resulted in a larger amount of preovulatory follicles with a greater capillary density within the theca layer of treated follicles [41].

During pregnancy, it is known that a certain degree of inflammation is required to ensure successful implantation of blastocyst into the maternal endometrium [42]. Plasma inflammatory cytokines were increased by leucine supplementation, which are indicators of the activated immune system. It is possible that the increased* LHR* concentration, together with IL-1*β* and IL-6 levels, could stimulate the expression of* VEGF* in luteal cells, which may influence concepts development at time of elongation and maternal recognition of pregnancy [6]. Besides, Plasma IgM and IgG were higher in leucine group. It might reflect a suitable physiology of maternal condition of its subsequent reproductive process. These results indicated that leucine supplementation might contribute to follicular development and improve oocyte quality.* Mucin-1* is decreased during the implantation phase, which provides access for embryo attaching to the uterine epithelium [43]. Nonetheless, available evidences showed that the reduction of Mucin-1 was required to permit embryo attachment in vivo rather than promoting this process [44, 45]. Intriguingly, in the present study, leucine decreased* Mucin-1* mRNA expression in ovary on day −1 of pregnancy. Yet, the identity of the mechanism remained elusive.

Oxidative stress has adverse effect on the developmental competence of oocyte [46], and antioxidant enzymes are proposed as potential indicators for monitoring of oocyte oxidative stress. The increase in SOD, CAT, and GSH-Px levels, as a result of leucine supplementation in this study, partly reflected amelioration in ovarian oxidative stress on day −1 of pregnancy.

In our previous study, arginine has been shown to enhance implantation sites, embryonic survival, and pregnancy outcome [47]. It has been argued that glutamine becomes the principal energy source when the glucose carbons are mainly used for synthesis of cell building blocks in proliferating cell [48]; notably, preimplantation conceptuses follow the aerobic glycolysis-glutaminolysis pattern [49]. Importantly, leucine, as one of the branched chain amino acids (BCAAs), is the substrate for the synthesis of glutamate and arginine in the metabolic pathway of amino acids [50, 51]. Correspondingly, in the current study, glutamine and arginine concentrations in plasma on day −1 of pregnancy were higher in leucine group. Maternal nutrition via mTOR signaling likely impacts on embryogenesis [52], which indicated that, through mTOR signaling pathway, leucine and glutamine might regulate intracellular protein turnover, therefore affecting the survival and growth of embryos and fetuses [47, 52].

## 5. Conclusions

In conclusion, our study not only points toward how leucine supplementation in premating diet increases litter homogeneity but also improves oocyte maturation and quality as well as embryo implantation. This is also the first study, to our knowledge, to demonstrate that leucine administration could reduce within-litter birth weight variation. Furthermore, leucine also increased the maternal antioxidant capacity and immune function, which could partly explain the better reproductive performance in leucine supplemented group. Our results provided much innovative information regarding approaches to mitigate variation of littermate birth weights, which could, in turn, improve neonatal survival.

## Figures and Tables

**Table 1:** Effect of dietary leucine supplementation on average daily feed intake during premating and pregnancy in primiparous SD rats.

Items	Control	0.6% leucine	*P* value
Average daily feed intake (g)			
Premating	16.87 ± 0.30	17.96 ± 0.38	0.314
Pregnancy	20.64 ± 0.32	21.74 ± 0.32	0.909

Value is the mean ± standard error of means of twelve replicates.

**Table 2:** Effect of dietary leucine supplementation on the reproductive performance in primiparous SD rats.

Items	Control	0.6% leucine	*P* value
Within-litter birth weight variation (%)	6.04 ± 0.01^*∗*^	5.30 ± 0.01	0.036
Number of live-born rats (*n*)	14.33 ± 0.28	16.00 ± 0.42	0.195
Average birth weight of live-born rats (g)	1.97 ± 0.01	2.12 ± 0.04^*∗∗*^	<0.01
Average weight of placentas (g)	0.45 ± 0.01	0.56 ± 0.03^*∗*^	0.021

Value is the mean ± standard error of means of twelve replicates; *∗* is means labeled with significant difference (*P* < 0.05); *∗∗* is means labeled with extreme difference (*P* < 0.01).

**Table 3:** Effect of dietary leucine supplementation on total number of implantation sites on day 7 of pregnancy in primiparous SD rats.

Items	Control	0.6% leucine	*P* value
Number of implantation sites (*n*)	15.90 ± 0.69	17.20 ± 0.49	0.321

Value is the mean ± standard error of means of ten replicates.

**Table 4:** Effect of dietary leucine supplementation on tissue weights on day −1 of pregnancy in SD rats.

Groups	Body weight	Absolute tissue weight		Percentage of BW	
		Ovary	Uterus	Ovary	Uterus
	g	mg	g	‰	
Control	271.57 ± 3.27	125.21 ± 0.01	0.39 ± 0.01	0.49 ± 0.01	1.42 ± 0.06
0.6% leucine	276.38 ± 4.48	141.33 ± 0.01^*∗*^	0.42 ± 0.04^*∗∗*^	0.52 ± 0.01	1.55 ± 0.16^*∗∗*^

Value is the mean ± standard error of means of ten replicates; *∗* is means labeled with significant difference (*P* < 0.05); *∗∗* is means labeled with extreme difference (*P* < 0.01).

**Table 5:** Effect of dietary leucine supplementation on plasma hormones in primiparous SD rats.

Items	Control	0.6% leucine	*P* value
Day −1 of pregnancy			
Insulin (uIU/mL)	18.44 ± 2.95^*∗*^	14.82 ± 1.25	0.017
IGF-1 (ng/mL)	157.90 ± 10.73	206.43 ± 33.99^*∗∗*^	≪0.01
Estradiol (pg/mL)	9.95 ± 0.97	10.76 ± 2.12^*∗*^	0.040
Progesterone (pg/mL)	406.24 ± 0.02	431.58 ± 0.01	0.493
LH (uIU/mL)	5.32 ± 0.10	5.87 ± 0.21^*∗*^	0.038
FSH (uIU/mL)	4.78 ± 0.99	6.37 ± 1.22	0.542
Day 7 of pregnancy			
Insulin (uIU/mL)	26.04 ± 1.79	16.40 ± 1.84	0.937
IGF-1 (ng/mL)	139.40 ± 8.39	229.70 ± 26.39^*∗∗*^	≪0.01
Estradiol (pg/mL)	5.91 ± 0.44	7.22 ± 1.35^*∗∗*^	≪0.01
Progesterone (pg/mL)	396.20 ± 0.01	412.97 ± 0.02^*∗*^	0.015
LH (uIU/mL)	5.14 ± 0.19	6.08 ± 0.13	0.369
FSH (uIU/mL)	6.84 ± 0.96	7.34 ± 1.44	0.305
Day 20 of pregnancy			
Insulin (uIU/mL)	22.47 ± 1.70	15.63 ± 1.42	0.617
IGF-1 (ng/mL)	195.43 ± 13.64	252.33 ± 30.15^*∗*^	0.038
Estradiol (pg/mL)	39.83 ± 3.17	43.12 ± 7.21^*∗*^	0.032
Progesterone (pg/mL)	429.65 ± 0.01	412.68 ± 0.02	0.424
LH (uIU/mL)	5.30 ± 0.28	5.60 ± 0.32	0.671
FSH (uIU/mL)	11.01 ± 1.16	4.67 ± 0.55^*∗*^	0.049

Value is the mean ± standard error of means of ten replicates; *∗* is means labeled with significant difference (*P* < 0.05); *∗∗* is means labeled with extreme difference (*P* < 0.01).

**Table 6:** Effect of dietary leucine supplementation on plasma and liver antioxidant enzyme activity and oxidant products on day −1 of pregnancy in SD rats.

Items	Control	0.6% leucine	*P* value
Plasma			
SOD (U/mL)	51.29 ± 2.60	63.02 ± 2.10^*∗∗*^	≪0.01
CAT (U/mL)	40.76 ± 3.36	48.66 ± 7.47^*∗*^	0.037
GSH-Px (U/mL)	848.58 ± 31.71	897.71 ± 43.12^*∗*^	0.032
MDA (nmol/ml)	5.05 ± 0.53	6.34 ± 0.65	0.56
Liver			
SOD (U/mL)	61.23 ± 5.97	76.66 ± 7.71	0.483
CAT (U/mL)	53.52 ± 3.20	58.66 ± 1.29^*∗*^	0.019
GSH-Px (U/mL)	611.10 ± 34.63	684.70 ± 20.78	0.170
MDA (nmol/ml)	2.90 ± 0.18	2.78 ± 0.11	0.143

Value is the mean ± standard error of means of nine replicates; *∗* is means labeled with significant difference (*P* < 0.05); *∗∗* is means labeled with extreme difference (*P* < 0.01).

**Table 7:** Effect of dietary leucine supplementation on the concentration of amino acids on day −1 and day 7 of pregnancy in SD rats.

Amino acid (ug/ml)	Control	Leucine	*P* value
Day −1 of pregnancy			
Cysteine	0.58 ± 0.14	0.41 ± 0.14	0.882
Phenylalanine	11.21 ± 0.49	13.17 ± 0.89	0.095
Alanine	32.99 ± 1.76	45.95 ± 3.88^*∗*^	0.027
Glycine	16.80 ± 1.52	21.06 ± 1.97	0.453
Glutamic acid	17.10 ± 0.73	21.54 ± 1.17	0.169
Glutamine	84.80 ± 4.82	89.30 ± 2.16^*∗*^	0.025
Methionine	5.60 ± 0.30	6.97 ± 0.46	0.217
Arginine	22.36 ± 1.94	24.51 ± 0.79^*∗*^	0.013
Lysine	106.49 ± 8.11	97.99 ± 5.88	0.353
Tyrosine	11.08 ± 1.03	12.10 ± 0.75	0.358
Leucine	15.89 ± 0.60	20.25 ± 1.42^*∗*^	0.017
Proline	15.24 ± 0.80	19.04 ± 0.86	0.810
Tryptophan	17.06 ± 0.77	19.62 ± 1.02	0.415
Serine	19.66 ± 1.26	23.99 ± 1.23	0.951
Threonine	30.58 ± 2.88	34.45 ± 2.90	0.981
Aspartic acid	0.54 ± 0.04	0.83 ± 0.15^*∗∗*^	≪0.001
Asparagine	6.91 ± 0.47	8.73 ± 0.41	0.728
Valine	18.79 ± 0.97	22.89 ± 1.27	0.427
Isoleucine	11.36 ± 0.47	14.09 ± 0.94	0.052
Histidine	6.87 ± 0.43	8.47 ± 0.74	0.118
Day 7 of pregnancy			
Cysteine	0.24 ± 0.06	0.35 ± 0.05	0.827
Phenylalanine	11.57 ± 0.52	11.85 ± 0.53	0.861
Alanine	40.13 ± 2.45	40.26 ± 1.21	0.091
Glycine	20.24 ± 1.14	20.10 ± 1.13	0.891
Glutamic acid	16.68 ± 1.06	16.88 ± 0.77	0.485
Glutamine	77.50 ± 3.62	75.84 ± 2.04	0.174
Methionine	7.04 ± 0.23	7.13 ± 0.12	0.090
Arginine	20.77 ± 0.60	23.81 ± 0.81	0.358
Lysine	79.78 ± 12.21	108.21 ± 7.97	0.325
Tyrosine	10.34 ± 0.62	11.56 ± 0.74	0.549
Leucine	18.44 ± 0.88	19.97 ± 0.58	0.347
Proline	17.47 ± 0.63	17.40 ± 0.58	0.919
Tryptophan	18.98 ± 1.11	16.65 ± 0.54	0.086
Serine	21.26 ± 0.96	20.75 ± 0.65	0.374
Threonine	33.12 ± 2.70	30.72 ± 1.40	0.116
Aspartic acid	0.71 ± 0.09	0.67 ± 0.03^*∗*^	0.019
Asparagine	7.31 ± 0.36	7.52 ± 0.35	0.917
Valine	21.84 ± 0.94	24.45 ± 0.90	0.968
Isoleucine	13.01 ± 0.67	13.93 ± 0.60	0.858
Histidine	7.89 ± 0.52	7.90 ± 0.30	0.185

Value is the mean ± standard error of means of eight replicates; *∗* is means labeled with significant difference (*P* < 0.05); *∗∗* is means labeled with extreme difference (*P* < 0.01).

## Data Availability

The data used to support the findings of this study are available from the corresponding author upon request.

## Abbreviations

BCAAs: Branched chain amino acids
CAT: Catalase
CYP17A1: Cytochrome p450 family 17 subfamily A polypeptide 1
CYP19A1: Cytochrome p450 family 19 subfamily A polypeptide 1
FSH: Follicle stimulating hormone
FSHR: Follicle stimulating hormone receptor
GSH-Px: Glutathione peroxidase
IGF-1: Insulin- like growth factor 1
IgA: Immunoglobulins A
IgG: Immunoglobulins G
IgM: Immunoglobulins M
IL-1*β*: Interleukins-1*β*
IL-6: Interleukins-6
LH: Luteinizing hormone
LHR: Luteinizing hormone receptor
MDA: Malondialdehyde
SOD: Superoxide dismutase
TNF *α*: Tumor necrosis factor *α*
VEGFA: Vascular endothelial growth factor A.

## Supplementary Materials

Supplementary Materials The specific primer sequences used in this study are shown in the Table S1.
