# Supplementary material for: Dietary Supplementation of Leucine in Premating Diet Improves the Within-Litter Birth Weight Uniformity, Antioxidative Capability, and Immune Function of Primiparous SD Rats
Source: Biomed Res Int. 2018 Apr 18;2018:1523147. doi: 10.1155/2018/1523147 (PMC5932505; doi:10.1155/2018/1523147)
Supplement: Supplementary Materials — The specific primer sequences used in this study are shown in the Table S1. [file 1523147.f1.pdf]

Online Supporting Material

Table S1. List of primer sequences

Gene	Forward (5'-3')	Reverse (5'-3')
β-actin[1]	TCCACCCGCGAGTACAACCTTCTT	CGACGAGCGCAGCGATATCGT
LHR	ATATTCAAGAGATGCACTGTGCAG	AAGCAGAGTGTCAATGGGAAATAG
FSHR	ACTGTGCATTCAACGGAA	GCCTCCATGAGGGTGACA
CYP17A1	ACTGAGGGTATCGTGGATGC	TCGAACTTCTCCCTGCACTT
CYP19A1	TCCTCAGCAGAGAAACTGGAAGA	CGTACAGAGTGACGGACATGGT
VEGFA	TGCACTGGACCCTGGCTTTAC	AGGGCTTCATCATTGCAGCAG
Mucin-1[2]	CCATCCTATGAGTGAATACCCTACCT	AAGAGAGACTGCTACTGCCATTACC

Supplemental Literatures Cited:

(1) Silva JF, Ocarino NM, Serakides R: **Luteal activity of pregnant rats with hypo-and hyperthyroidism.** *Journal of ovarian research* 2014, **7:**75.

(2) LEE D-S, UETA YY, XUAN X, IGARASHI I, FUJISAKI K, SUGIMOTO C, TOYODA Y, SUZUKI H: **Expression patterns of the implantation-associated genes in the uterus during the estrous cycle in mice.** *Journal of Reproduction and Development* 2005, **51:**787-798.
